# Eye-Tracking-Based Evaluation of Visual Search Efficiency in Simulated VR Menu Interfaces: Effects of Card Layout Structure and Target Spatial Quadrant

**DOI:** 10.3390/s26123652

**Published:** 2026-06-08

**Authors:** Jing Zhang, Yanxu Zhou, Chenyu Xu, Yulin Zhu, Jingjing Li, Jing Li

**Affiliations:** College of Furnishings and Industrial Design, Nanjing Forestry University, Nanjing 210037, China; zhouyanxu@njfu.edu.cn (Y.Z.); xuchenyuuu@njfu.edu.cn (C.X.); zhuyulin0828@njfu.edu.cn (Y.Z.); lijingjingg@njfu.edu.cn (J.L.); lijingnjfu@njfu.edu.cn (J.L.)

**Keywords:** virtual reality interface, card layout, visual search, eye-tracking sensor, spatial attention, grid-based design, gallery-based design, desktop-based simulation

## Abstract

Understanding how interface layout influences visual search performance is important for optimizing virtual reality (VR) interfaces. This study investigated visual search efficiency and gaze behavior in simulated VR menu interfaces using a screen-based eye-tracking experiment. To enable controlled measurement of gaze behavior and isolate layout-driven perceptual effects, the interfaces were evaluated using a desktop-based VR simulation. The experiment examined two independent variables: menu layout structure and target spatial quadrant. Two representative VR menu layout structures were compared: a grid-based layout arranged as a 4 × 4 matrix and a gallery-based layout consisting of four large and twelve small cards, forming a size-based visual hierarchy. Target locations were distributed across four spatial quadrants: lower-left, lower-right, upper-left, and upper-right. Participants (*N* = 39) completed visual search tasks while accuracy (ACC), reaction time (RT), and eye-tracking metrics, including total visit duration (TVD) and total fixation count (TFC), were recorded. The results showed that the gallery-based layout supported more efficient visual search than the grid-based layout, as reflected in shorter RTs, reduced overall TVD, and lower overall TFC. Behavioral and eye-tracking analyses also revealed systematic spatial asymmetries, with the upper-right quadrant showing the fastest responses and reduced gaze-based search effort. Importantly, the advantage of the gallery-based layout was most consistent in the upper-right quadrant, indicating that layout structure and target spatial quadrant jointly shaped visual search efficiency. Gaze-distribution heatmaps provided qualitative visual support for these patterns. These findings provide early-stage perceptual evidence for optimizing layout hierarchy in simulated VR menu interfaces and demonstrate the value of screen-based eye-tracking sensors as quantitative tools for evaluating attentional allocation before further validation in immersive HMD-based VR environments.

## 1. Introduction

With the continued development and widespread adoption of virtual reality (VR) technologies, VR interfaces have become primary environments through which users access and interact with virtual content, especially as eye tracking becomes increasingly available in head-mounted displays [1]. VR menu interfaces function as critical entry points that guide users in selecting and accessing subsequent actions. VR menu interfaces are characterized by stereoscopic depth cues (e.g., binocular disparity), spatially distributed 3D elements for pointing/selection, a wide field of view (FOV), and a reliance on coordinated head–eye exploration [2,3,4]. Based on these characteristics, users must rapidly locate and recognize visual elements in three-dimensional space. Compared with conventional 2D interfaces, VR menus may impose additional demands on spatial attention and cognitive resources due to increased exploration requirements and head–gaze coordination [5]. Although fully immersive VR systems allow direct measurement of head–gaze coordination through integrated eye trackers and inertial measurement units, screen-based eye tracking provides a controlled and reproducible approach for isolating layout-driven visual attention effects during early-stage interface evaluation. Prior studies in graphical user interfaces and display design have shown that interface layout significantly affects visual search performance, fixation allocation, and cognitive efficiency [6,7,8,9].

**Figure 1:**
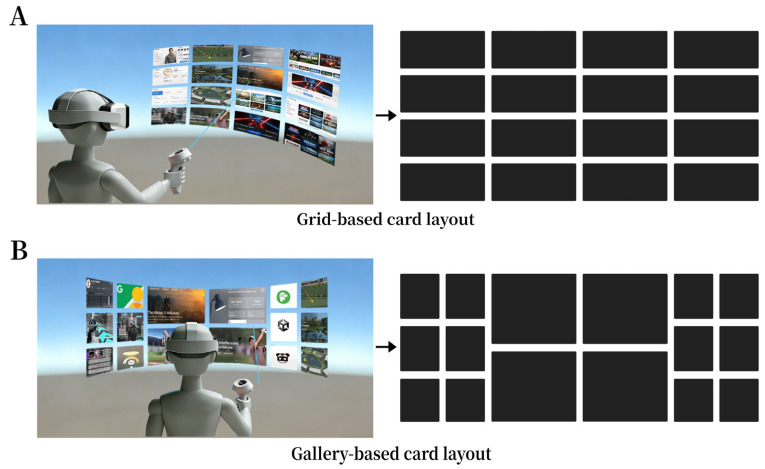
Schematic examples of two representative card-based VR menu layout structures: (**A**) grid-based layout and (**B**) gallery-based layout.

However, in the context of VR, menu layouts exhibit diverse forms in practice, and systematic investigations specifically focusing on layout structure in VR menu interfaces remain limited. Among various VR menu designs, card-based layouts are often adopted in VR menu design because they support modular information presentation and rapid item selection. Given the limited standardized academic taxonomy of card-based VR menu layouts, this study abstracted two representative organization patterns from common card-based content-browsing interfaces in practical VR/MR applications. The grid-based layout consists of uniformly sized menu items arranged in a regular matrix, whereas the gallery-based layout uses menu items with differentiated sizes to create a hierarchical visual structure. These two layouts were selected to represent a uniform matrix organization and a non-uniform, size-hierarchical organization. Figure 1 presents schematic examples of these two layout structures. Therefore, understanding visual search and attentional mechanisms in VR menu interfaces is crucial for optimizing interface layout design.

While prior research has explored gaze-based interaction and spatial cognition in VR, relatively few studies have systematically compared how different VR menu layouts affect visual search performance and attention allocation. Existing studies have primarily examined interaction techniques or input modalities, rather than layout-driven perceptual effects [10,11]. In addition, prior studies have reported spatial asymmetries in attention, including faster searches in the upper or right regions of the visual field, in 2D displays and non-immersive 3D environments [12,13,14]. However, it remains unclear whether these biases persist in VR menu interfaces and how layout structure interacts with spatial direction to influence search behavior. Although eye tracking has been increasingly applied in VR research [1,3,15], few studies have systematically examined how layout structure influences gaze behavior in VR menu interfaces using controlled experimental paradigms. Therefore, it is necessary to further investigate how layout structure and spatial direction interact to shape visual search behavior.

To address these gaps, this study investigates how two typical VR menu layout designs affect users’ visual search behavior using a desktop-based visual simulation approach. The present study does not aim to replace fully immersive VR evaluation. Instead, it focuses on an early-stage perceptual evaluation paradigm in which layout-related visual attention effects can be isolated under controlled viewing conditions. We presented static rendered images of VR card menu layouts on a computer screen, preserving the spatial organization and visual hierarchy of immersive menus in a controlled setting. This desktop-based simulation approach was informed by prior comparisons of virtual reality, desktop-based 3D, and 2D experimental conditions using behavioral and gaze-related measures [16]. This approach can provide a methodological reference and comparative basis for subsequent research that integrates head-mounted eye-tracking sensors in fully immersive environments. Accordingly, this study treats layout structure and target spatial quadrant as two key experimental factors and evaluates their effects using behavioral performance and eye-tracking measures. Specifically, this study addresses three research questions:

RQ1: How does menu layout structure, specifically grid-based versus gallery-based layouts, affect visual search performance and gaze behavior?

RQ2: Do spatial asymmetries emerge across target spatial quadrants in simulated VR menu interfaces?

RQ3: Does layout structure interact with target spatial quadrants in shaping visual search efficiency?

From a measurement perspective, the study also examines whether screen-based eye-tracking metrics can serve as sensitive indicators for evaluating layout-induced attention differences in VR interface design. By combining behavioral and eye-tracking data, this study provides early-stage perceptual evidence on how layout configuration and spatial organization influence users’ visual attention, contributing to a deeper understanding of visual search mechanisms and eye-tracking-based evaluation methods in VR interface design.

## 2. Related Work

### 2.1. VR Menu Layout

Prior studies on VR interfaces and menus have largely emphasized interaction techniques and input modalities, such as gaze-, head-, and controller-based targeting, and have compared their efficiency and usability under different task demands [17,18,19]. Although these works provide important guidance for interaction design, they often treat menu layout as a background condition rather than a primary manipulation, leaving the role of layout in perceptual guidance relatively underexplored in VR menu search. In sensor-based interface evaluation, layout should not be viewed merely as a cosmetic choice; rather, it can modulate attentional resource allocation and be reflected in sensor-captured gaze behavior. In parallel, a growing body of eye-tracking research in graphical user interfaces (e.g., desktop and mobile interfaces) and display systems demonstrates that layout structure and information organization can directly modulate search efficiency and gaze behavior. For example, manipulating layout and display modes in guidance interfaces yields measurable differences in task performance and visual behavior [20], and layout choices for head-worn augmented reality interfaces can alter usability outcomes and task success [21]. Prior studies have also shown that layout-related design factors, including visual grouping cues, spatial layout coding, and graphical layout structure, can influence menu-search performance, visual search efficiency, response time, and fixation-related search strategies [22,23,24]. Beyond conventional graphical user interfaces, evidence from extended reality interfaces (including augmented and mixed reality systems) suggests that spatial interface organization may influence attentional guidance in immersive contexts [25].

These findings are consistent with the Guided Search framework, which suggests that visual features such as size, salience, and spatial organization can guide attention toward prioritized regions during visual search [26]. In gallery-based card layouts, larger cards may function as perceptual anchors that help users organize the search space and reduce unnecessary visual exploration. These studies suggest that layout structure can act as an attentional guide rather than merely a visual presentation format. However, in VR menu interfaces, layout has often been treated as a background design feature, and few studies have isolated card layout structure as an independent experimental factor. In particular, limited evidence is available on whether size-based visual hierarchy in gallery-based VR menu layouts can improve search efficiency and alter eye-tracking measures of visual attention.

### 2.2. Spatial Location

In addition to layout structure, another important factor influencing visual search performance in interface design is the spatial distribution of information. Previous studies have shown that visual attention is not evenly distributed across the visual field, but instead exhibits systematic spatial asymmetries in visual processing and attentional allocation [27]. These asymmetries suggest that target location may influence search efficiency. However, in structured interface search tasks, spatial biases may also be modulated by top-down factors, including reading habits, interface conventions, task goals, and learned scanning strategies. Therefore, it remains necessary to examine whether previously reported spatial asymmetries persist in VR-like menu interfaces and how they interact with layout-driven visual hierarchy.

#### 2.2.1. Vertical and Horizontal Asymmetries

For vertical asymmetries, prior work has shown that upper and lower visual fields are functionally specialized and can exhibit different efficiency profiles depending on task demands [28]. Empirical studies have reported an upper visual-field advantage in target detection tasks, reflected in faster response times and more efficient visual search [29]. For horizontal asymmetries, recent evidence from a controlled orientation-discrimination task suggests that a right-visual-field advantage may emerge in behavioral performance, including reaction time [30]. These asymmetries may also be shaped by top-down and experience-related factors. For example, native reading direction can influence lateral biases in visual perception, and experimental manipulations of attentional balance may modulate left–right attentional asymmetries [31,32]. It should be noted that spatial asymmetries reported in low-level visual detection or discrimination tasks may not directly translate to structured interface search. In interface environments, biological constraints on visual performance may interact with top-down factors, including reading habits, interface conventions, task goals, and learned scanning strategies. Therefore, the direction and magnitude of spatial advantages in VR-like menu interfaces require empirical examination rather than direct inference from basic visual-field studies.

#### 2.2.2. Evidence from Eye-Tracking and VR Contexts

Eye-tracking technology provides an objective means of capturing gaze behavior and visual attention, and has been widely applied in human–computer interaction, visual perception, and VR-related research [1,33]. In visual search and interface-evaluation studies, gaze-based measures have been used to quantify gaze allocation, fixation distribution, and attentional processes during task performance [6,7,8,9]. From a sensor-based evaluation perspective, eye tracking provides objective and time-resolved measurements of gaze allocation, making it suitable for detecting subtle differences in visual attention that may not be fully captured by behavioral measures alone. In particular, measures such as total visit duration (TVD) and total fixation count (TFC) provide useful indicators of how visual attention is distributed across interface regions, making them suitable for evaluating the effects of layout structure and spatial location on visual search behavior. TVD reflects the amount of time users allocate to specific interface regions, whereas TFC reflects the frequency of visual sampling during search. Together, these metrics can capture both sustained processing and search effort. In immersive and VR-like settings, eye-tracking has been used to examine gaze allocation during visual search and spatial attention tasks across extended or simulated visual environments [1,3,5,15]. Eye-tracking evidence further shows that fixation distribution during visual search varies across different regions of the display, rather than being spatially uniform [34]. In addition, prior research in VR interaction contexts has shown that target location can influence task performance, suggesting that spatial position may influence interaction efficiency in immersive environments [35].

However, these studies have primarily focused on interaction techniques or spatial positioning, rather than layout-based visual search. These findings highlight the need to examine whether spatial asymmetries observed in non-VR interface contexts generalize to VR environments, where interaction and exploration strategies differ. Therefore, it remains unclear whether previously reported spatial asymmetries persist in VR menu interfaces and how layout structure interacts with spatial location to influence visual search behavior. In particular, few studies have jointly manipulated card layout structure and target quadrant within a controlled experimental framework while combining behavioral measures with eye-tracking metrics. These gaps motivate the present study, which compares grid-based and gallery-based card layouts across four target quadrants using behavioral and eye-tracking measures.

## 3. Methods

**Figure 2:**
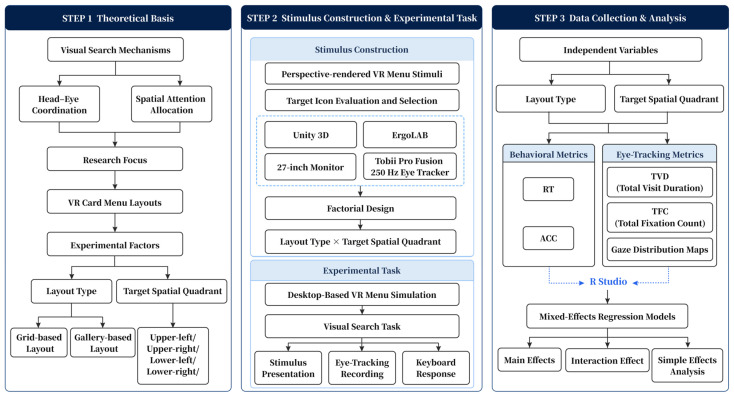
Overview of the theoretical basis, experimental design, and eye-tracking-based data analysis pipeline.

This study employed a controlled, desktop-based visual simulation paradigm to investigate how VR menu layout structure (grid-based vs. gallery-based card layout) and target spatial quadrant (lower-left, lower-right, upper-left, and upper-right) shape visual search performance and attentional allocation. Desktop-based VR simulations have been used as a rigorous early-stage approach for isolating perceptual and attentional mechanisms under high experimental control, while still retaining key spatial properties of immersive interfaces. Figure 2 summarizes the theoretical basis, eye-tracking-based experimental design, dependent measures, and statistical analysis pipeline.

### 3.1. Participants

A total of 40 participants were initially recruited from a local university community (18 males and 22 females; mean age M = 21.93 years, SD = 1.14). One participant was excluded because of incomplete eye-tracking data collection, resulting in a final analytical sample of 39 participants. All participants reported normal or corrected-to-normal vision and no history of neurological or visual disorders. Written informed consent was obtained before the experiment. The study protocol followed institutional ethical standards for research with human participants.

### 3.2. Experimental Design

The experiment adopted a 2 × 4 within-subject factorial design with two independent variables: menu layout structure (grid-based card layout vs. gallery-based card layout) and target spatial quadrant (lower-left, lower-right, upper-left, and upper-right). Each participant completed visual search trials in all eight conditions formed by the combination of layout type and target quadrant. Target locations were counterbalanced and equally distributed across the four quadrants, ensuring that each quadrant was presented the same number of times for each layout type. The target position within a given quadrant was randomized across cards to minimize positional learning effects. This design allowed us to test both the main effect of menu layout structure and the effect of spatial position on visual search efficiency, as well as their potential interaction.

### 3.3. Experimental Materials

**Figure 3:**
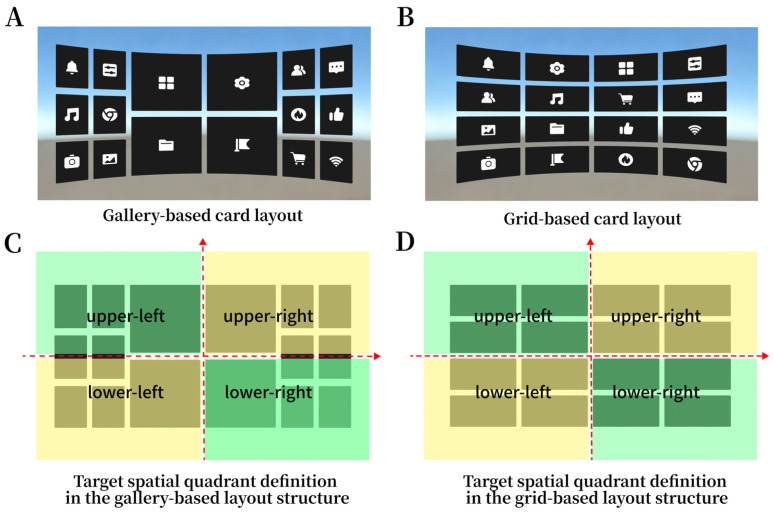
Examples of the experimental VR menu stimuli and target spatial quadrant definitions. (**A**) Gallery-based card layout rendered on a shallow cylindrical display surface; (**B**) grid-based card layout rendered on a shallow cylindrical display surface; (**C**) target spatial quadrant definition in the gallery-based layout structure; and (**D**) target spatial quadrant definition in the grid-based layout structure. The quadrant diagrams in (**C**,**D**) are schematic illustrations used to define target spatial locations.

To simulate a virtual reality (VR) interface under controlled laboratory conditions, static rendered images of two VR menu layout types were created using Unity 3D (Version 2021.3.20f1; Unity Technologies, San Francisco, CA, USA). Each menu layout represented a typical user’s view of a VR card menu environment. Two layout configurations were created: a grid-based card layout, where sixteen equally sized cards were arranged in a 4 × 4 matrix, and a gallery-based card layout, where sixteen cards included four large cards and twelve small cards, forming a non-uniform visual structure with size variation, as shown in Figure 3A,B. In this study, the term “gallery-based layout” is used as an operational and descriptive term to refer to this representative size-hierarchical card configuration. It denotes a non-uniform card-based organization in which card-size variation creates visual salience and potential attentional anchors, rather than a standardized taxonomy of VR menu layouts. Target spatial quadrants were defined as lower-left, lower-right, upper-left, and upper-right, as illustrated in Figure 3C,D.

To approximate the spatial presentation form of VR menu interfaces, the two-dimensional menu layouts were mapped onto a shallow cylindrical surface before rendering. Specifically, the virtual information panel was configured as a slightly concave cylindrical canvas facing the observer, with a curvature radius of 3.0 m and a horizontal angular span of approximately 40°. The rendered menu stimulus subtended a visual angle of approximately 40° horizontally and 22° vertically at the fixed viewing distance. This low-curvature setting preserved the original planar layout structure while simulating the mildly curved display form often used to present two-dimensional information in VR environments. Previous studies have shown that display type, field of view, and curvature may affect visual search, text readability, and pointing performance in VR or display-based environments [36,37,38]. To avoid introducing display curvature as a confounding factor, the same curvature setting was applied to both layout conditions; curvature itself was therefore not treated as an independent variable in the present study.

The experimental environment used Unity 3D’s default Procedural Skybox as the background to provide a low-interference visual reference. The sky tint was set to a medium gray-blue color (Hex: #7392B7), and the ground color was set to dark gray (Hex: #474542), creating a simple outdoor-like lighting impression with a horizon reference. These background settings were kept constant across all stimuli.

Each layout condition included 12 trials, resulting in a total of 24 trials per participant. For each layout type, target locations were counterbalanced and equally distributed across the four quadrants, with three repetitions per quadrant. Within each quadrant, the target position was varied across different cards to reduce positional learning effects.

Each card displayed one icon to ensure comparable visual complexity across layouts. To control the potential influence of icon characteristics on participants’ cognition, all target icons were selected from a pre-validated icon set developed in a prior pilot study. Specifically, 45 candidate icons were evaluated by 33 participants using three 5-point Likert scales assessing visual complexity, familiarity, and concreteness. Based on the rating results, 16 icons were selected according to the predefined criteria of low visual complexity, high familiarity, and high concreteness. Across the selected icon means, the selected icons showed low visual complexity (M = 1.73, SD = 0.09), high familiarity (M = 4.36, SD = 0.05), and high concreteness (M = 4.35, SD = 0.06). One-way ANOVA results revealed no significant differences among the selected icons in concreteness, F(15,512) = 0.329, *p* = 0.992, visual complexity, F(15,512) = 0.549, *p* = 0.912, or familiarity, F(15,512) = 0.190, *p* > 0.999. All icons were placed at the center of the information cards. The icon size was standardized at 64 × 64 pixels, and the icon color was set to white (Hex: #FFFFFF) to reduce potential color-related effects.

Based on this design, the critical manipulation was layout structure, namely the uniform grid layout versus the non-uniform gallery layout, rather than differences in the number of elements, icon semantics, icon color, background setting, or display curvature. Although the experiment was conducted on a desktop monitor rather than with a head-mounted display (HMD), the static rendered images were designed to preserve the spatial organization, card layout structure, shallow curvature, and basic depth cues of VR menu interfaces. Prior work has suggested that desktop- or screen-based implementations can serve as controlled approximations for investigating visual search and attentional mechanisms in VR-related tasks, particularly when high experimental control is required in early-stage evaluation [5,39]. Accordingly, the present desktop-based simulation approach was used as a controlled first step before future validation in fully immersive VR settings. This methodological choice was intended to prioritize experimental control and isolate layout-driven visual attention effects, rather than to reproduce the full perceptual and interaction experience of immersive HMD-based VR.

### 3.4. Experimental Procedure

**Figure 4:**
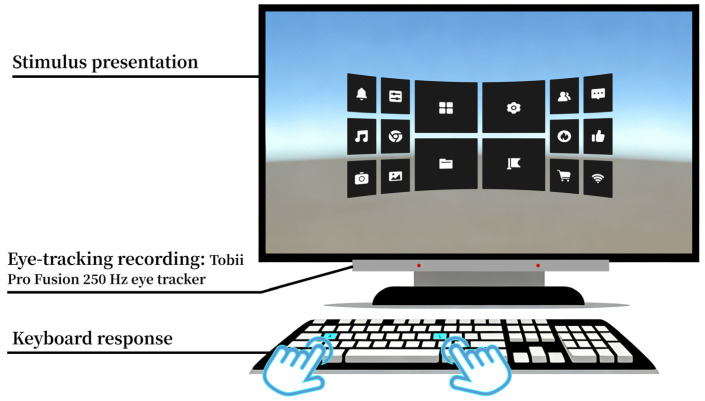
Experimental setup showing stimulus presentation, eye-tracking recording, and keyboard response.

The experiment was conducted on a desktop computer equipped with an HP 27-inch monitor (HP Inc., Palo Alto, CA, USA; 1920 × 1080 pixels, 60 Hz). Eye movements were recorded using a Tobii Pro Fusion 250 eye tracker (Tobii Pro AB, Danderyd, Sweden) with a sampling rate of 250 Hz, and the viewing distance was maintained at approximately 60 cm. Both behavioral and eye movement data were recorded using ErgoLAB (version 3.3.5; Kingfar Technologies Inc., Beijing, China).The experimental setup, including stimulus presentation, eye-tracking recording, and keyboard response, is shown in Figure 4.

**Figure 5:**
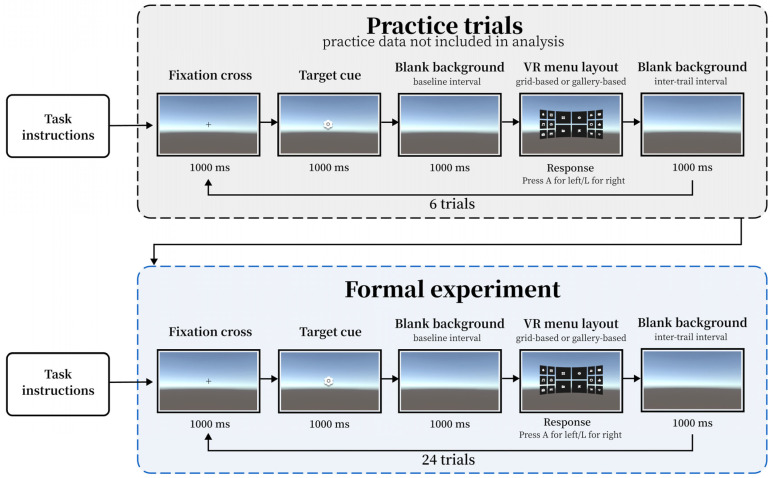
Trial sequence of the visual search task.

Participants were tested individually in a quiet laboratory. They were seated comfortably with their gaze aligned to the screen center at an approximately fixed viewing distance (≈60 cm). Before the formal session, a nine-point calibration was performed for each participant, and recalibration was conducted whenever tracking quality degraded to ensure stable eye-tracking recordings, consistent with recent screen-based eye-tracking interface-search studies [6,40]. Prior to data collection, participants completed six practice trials to familiarize themselves with the trial sequence and the response mapping; practice data were not included in the analyses. In the formal session, each participant completed 24 trials (12 trials per layout type), with targets appearing equally across the four quadrants (three repetitions per quadrant). First, a central fixation cross (“+”) was presented for 1000 ms to standardize the initial gaze position and reduce carryover effects. The fixation cross then disappeared and the default background remained on screen for 1000 ms as a baseline interval. Next, a target cue indicating the icon to be searched was presented for 1000 ms. Immediately after the cue offset, the VR menu screenshot (grid-based or gallery-based layout) appeared and remained visible until a response was made. Participants were instructed to indicate whether the target icon appeared on the left or right side of the display by pressing “A” for left and “L” for right. The left/right response mapping was used to obtain a rapid behavioral index of target localization while avoiding potential confounds from mouse movement, pointing accuracy, controller operation, or dwell-time confirmation. This response mode was therefore intended to isolate visual search and localization processes, rather than to fully reproduce natural VR selection behavior. Following the response, the menu display was replaced by the default background for 1000 ms to reduce visual persistence and minimize residual effects before the next trial. The complete trial sequence, including fixation, baseline interval, target cue, menu search, keyboard response, and inter-trial interval, is illustrated in Figure 5. The full session lasted approximately 25 min per participant, including calibration and short breaks to mitigate visual fatigue and maintain tracking quality.

### 3.5. Data Processing and Statistical Modeling

Dependent measures included response accuracy (ACC), reaction time (RT), and two primary eye-movement indices capturing search efficiency and attentional allocation: total visit duration (TVD) and total fixation count (TFC). ACC was used to evaluate task correctness, whereas RT was used as the primary behavioral indicator of visual search efficiency. TVD was used to quantify the cumulative duration of visual attention allocated to the interface during search, whereas TFC was used to quantify the frequency of visual sampling. For both TVD and TFC, the summed values across predefined AOIs were used as the primary gaze-based measures. Gaze-distribution heatmaps were additionally generated to visualize spatial allocation of attention across layout structures and target quadrants.

Areas of interest (AOIs) were defined to cover the entire visible menu card region in each stimulus. The overall menu AOI included all information cards and was used to extract gaze-based measures during the visual search period. For spatial analysis, target locations were categorized according to the four predefined quadrants: lower-left, lower-right, upper-left, and upper-right. Eye-tracking metrics were calculated from the onset of the menu stimulus to the participant’s response. All gaze-based measures, including TVD, TFC, and gaze-distribution heatmaps, were extracted or generated using ErgoLAB 3.3.5.

Behavioral and eye-tracking data were processed and analyzed using RStudio (version 2025.05.0+496). Mixed-effects models were implemented using the lme4 package (Version 2.0-1) [41], and post hoc comparisons were conducted using the emmeans package (Version 1.8.5) [42]. To account for the repeated-measures structure of the within-subject design, all inferential analyses were conducted using mixed-effects models rather than simple independent-sample comparisons. Statistical significance was evaluated at α = 0.05. ACC, coded as a binary outcome, was analyzed using logistic mixed-effects models (GLMMs), whereas RT, TVD, and TFC were analyzed using linear mixed-effects models (LMMs), following established recommendations for categorical and continuous repeated-measures data [43,44]. For all models, menu layout structure, target spatial quadrant, and their interaction were specified as fixed effects, and participant was included as a random intercept. Incorrect trials were excluded from RT and eye-tracking analyses. For correct trials, RT outliers were removed using a participant-level 3 × median absolute deviation (MAD) criterion. Model comparisons were conducted using likelihood-ratio tests (χ^2^) and changes in Akaike Information Criterion (ΔAIC). For significant interactions, follow-up simple-effect comparisons were conducted using estimated marginal means. Adjusted *p* values were reported after Bonferroni correction across the four quadrant-specific layout comparisons.

## 4. Results

### 4.1. Data Screening

After applying the data-processing criteria described in Section 3.5, invalid trials were excluded prior to analysis. For the RT and eye-tracking analyses, only correct trials were retained (the overall error rate was 1.28%). RT outliers were then excluded using a participant-level 3 × median absolute deviation (MAD) criterion, resulting in the removal of 11.3% of correct trials.

### 4.2. Behavioral Data

Means and standard deviations of accuracy and reaction time across experimental conditions are presented in Table 1. Inferential results for behavioral and eye-tracking measures are reported below based on the mixed-effects modeling procedure described in Section 3.5.

**Figure 6:**
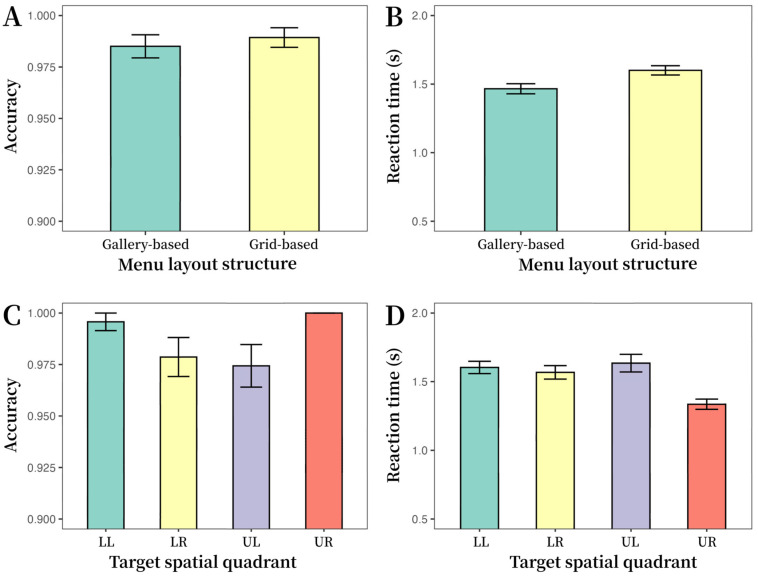
Behavioral descriptive performance across menu layout structures and target spatial quadrants. (**A**) Accuracy by menu layout structure; (**B**) reaction time by menu layout structure; (**C**) accuracy by target spatial quadrant; and (**D**) reaction time by target spatial quadrant. Error bars represent standard errors. LL = lower-left; LR = lower-right; UL = upper-left; UR = upper-right.

Descriptive behavioral performance across menu layout structures and target spatial quadrants is shown in Figure 6. Accuracy was high across all conditions, indicating that participants performed the visual search task reliably. The logistic mixed-effects analysis showed that adding menu layout structure did not improve model fit (ΔAIC = +1.66, LLR χ^2^(1) = 0.34, *p* = 0.559). By contrast, target spatial quadrant significantly improved model fit (ΔAIC = −5.39, LLR χ^2^(3) = 11.39, *p* = 0.010), suggesting that response accuracy differed slightly across target locations. The interaction between menu layout structure and target spatial quadrant was not significant (ΔAIC = +4.75, LLR χ^2^(3) = 1.25, *p* = 0.740). Given the near-ceiling accuracy, ACC was interpreted primarily as a task-validity indicator, with RT and eye-tracking measures providing more sensitive indices of search efficiency.

Reaction time showed clear sensitivity to both layout structure and target location. Including menu layout structure significantly improved model fit (ΔAIC = −5.30, LLR χ^2^(1) = 7.25, *p* = 0.007), indicating that visual search speed differed between the grid-based and gallery-based layouts. Target spatial quadrant provided an additional improvement in model fit (ΔAIC = −20.30, LLR χ^2^(3) = 26.33, *p* < 0.001), confirming that response speed varied across interface regions. The interaction between menu layout structure and target spatial quadrant was also significant (ΔAIC = −5.20, LLR χ^2^(3) = 11.19, *p* = 0.011), indicating that the layout effect was not uniform across quadrants.

Descriptively, under the present VR-like menu stimuli, responses were fastest when targets appeared in the upper-right quadrant of the gallery-based layout (M = 1.178 s) and slowest when targets appeared in the lower-left quadrant of the grid-based layout (M = 1.710 s). This pattern was consistent with the significant layout structure × target spatial quadrant interaction.

Bonferroni-corrected simple-effect comparisons are summarized in Table 2. The comparisons showed that the gallery-based layout produced significantly shorter RTs than the grid-based layout only in the upper-right quadrant (estimate = −0.313 s, SE = 0.092, t(785) = −3.39, *p*_adj = 0.003). The corresponding layout differences in the other three quadrants were not significant after correction (all *p*_adj ≥ 0.110). A numerical advantage for the gallery-based layout was also observed in the lower-left quadrant, but this effect did not survive Bonferroni correction.

**Figure 7:**
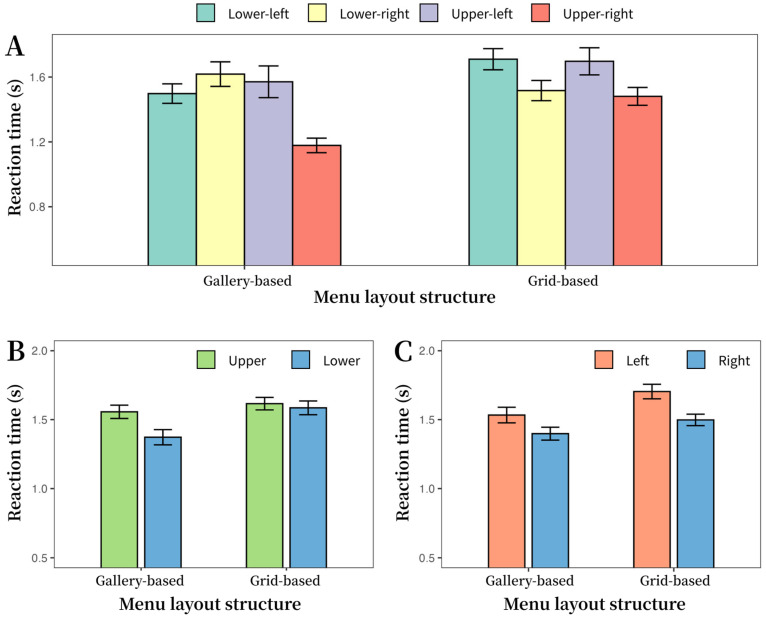
Reaction time patterns across menu layout structures, target quadrants, and spatial directions. (**A**) Mean reaction time for each layout-by-quadrant condition; (**B**) mean reaction time by vertical region (upper vs. lower), grouped by menu layout structure; and (**C**) mean reaction time by horizontal region (left vs. right), grouped by menu layout structure. Error bars represent standard errors. UL = upper-left; UR = upper-right; LL = lower-left; LR = lower-right.

As shown in Figure 7B,C, follow-up directional analyses were conducted by collapsing target quadrants into vertical regions (upper vs. lower) and horizontal regions (left vs. right). The vertical-region analysis showed that upper targets were associated with faster responses than lower targets (upper: M = 1.47 s; lower: M = 1.58 s; ΔAIC = −3.20, LLR χ^2^(1) = 5.25, *p* = 0.022), and the layout structure × y-location interaction did not reach significance (*p* = 0.090). The horizontal-region analysis showed a stronger left–right difference, with faster responses for right-side targets than for left-side targets (right: M = 1.44 s; left: M = 1.61 s; ΔAIC = −11.70, LLR χ^2^(1) = 13.71, *p* < 0.001), while the layout structure × x-location interaction was not significant (*p* = 0.533). These results suggest that the upper-right advantage reflected the combined contribution of upper-region and right-side facilitation.

### 4.3. Eye-Tracking Measures

**Figure 8:**
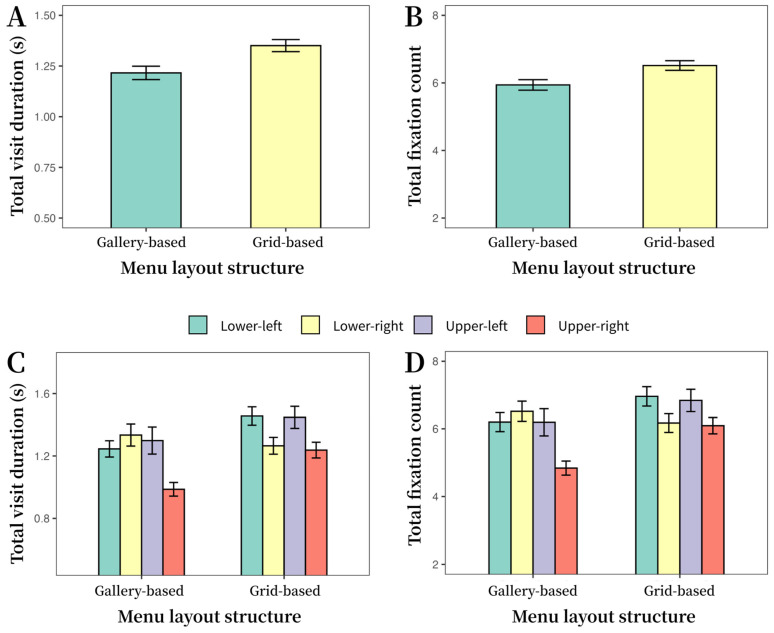
Eye-tracking condition-level patterns across menu layout structures and target spatial quadrants. (**A**) Total visit duration (TVD) by menu layout structure; (**B**) total fixation count (TFC) by menu layout structure; (**C**) TVD for each layout-by-quadrant condition; and (**D**) TFC for each layout-by-quadrant condition. Error bars represent standard errors. UL = upper-left; UR = upper-right; LL = lower-left; LR = lower-right.

Descriptive statistics for the eye-tracking measures are presented in Table 3, and the corresponding condition-level patterns are illustrated in Figure 8. Overall, the eye-tracking measures showed patterns broadly consistent with the behavioral RT results under the desktop-based simulated VR conditions.

A significant pattern was first observed for total visit duration (TVD). Including menu layout structure significantly improved model fit (ΔAIC = −7.90, LLR χ^2^(1) = 9.96, *p* = 0.002), indicating that visual attention duration differed between the two layout structures. Target spatial quadrant also improved model fit (ΔAIC = −23.60, LLR χ^2^(3) = 29.55, *p* < 0.001), showing that the duration of visual attention varied across target locations. The menu layout structure × target spatial quadrant interaction was also significant (ΔAIC = −5.20, LLR χ^2^(3) = 11.19, *p* = 0.011), suggesting that the layout effect on sustained visual attention depended on the target quadrant. Bonferroni-corrected simple-effect comparisons showed that the gallery-based layout produced significantly shorter visit durations than the grid-based layout in the lower-left quadrant (estimate = −0.200 s, SE = 0.078, t(784) = −2.57, *p*_adj = 0.042) and the upper-right quadrant (estimate = −0.277 s, SE = 0.078, t(784) = −3.54, *p*_adj = 0.002). No significant layout differences were observed in the lower-right or upper-left quadrants after correction (both *p*_adj ≥ 0.602).

For total fixation count (TFC), including menu layout structure significantly improved model fit (ΔAIC = −5.90, LLR χ^2^(1) = 7.91, *p* = 0.005), indicating that the number of fixations differed between the two layout structures. Target spatial quadrant provided a further improvement in model fit (ΔAIC = −16.50, LLR χ^2^(3) = 22.51, *p* < 0.001), suggesting that fixation count varied across target locations. The interaction between menu layout structure and target spatial quadrant was also significant (ΔAIC = −3.80, LLR χ^2^(3) = 9.73, *p* = 0.021), indicating that the layout effect on fixation count was not uniform across quadrants. Bonferroni-corrected simple-effect comparisons further showed that the gallery-based layout produced significantly fewer fixations than the grid-based layout only in the upper-right quadrant (estimate = −1.339, SE = 0.386, t(785) = −3.47, *p*_adj = 0.002). The corresponding layout differences in the other three quadrants were not significant after correction (all *p*_adj ≥ 0.224). These simple-effect results are summarized in Table 4.

**Figure 9:**
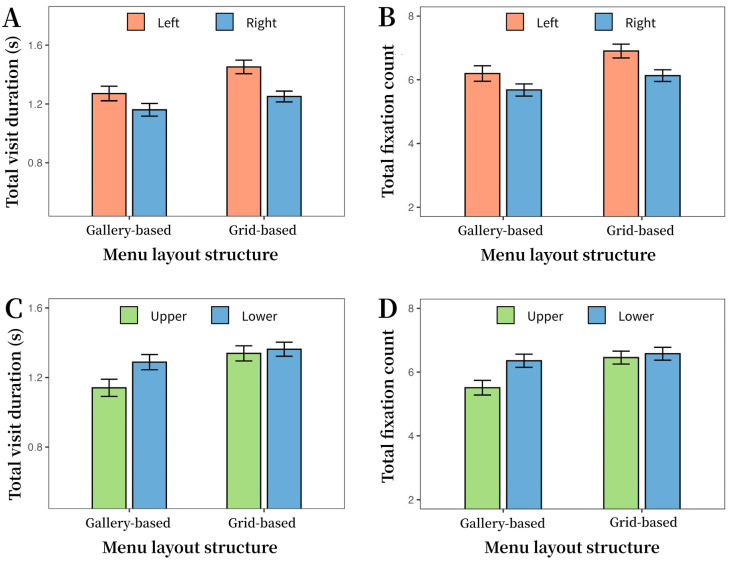
Eye-tracking patterns across spatial directions. (**A**) TVD by horizontal region; (**B**) TFC by horizontal region; (**C**) TVD by vertical region; (**D**) TFC by vertical region. Error bars represent standard errors.

Directional follow-up analyses further showed that upper and right-side targets required shorter visit durations and fewer fixations than lower and left-side targets. For TVD, upper targets yielded shorter visit durations than lower targets (upper: M = 1.23 s, SE = 0.052; lower: M = 1.32 s, SE = 0.052; *p* = 0.023), and right-side targets yielded shorter visit durations than left-side targets (right: M = 1.19 s, SE = 0.053; left: M = 1.36 s, SE = 0.053; *p* < 0.001). For TFC, upper targets required fewer fixations than lower targets (upper: M = 5.92, SE = 0.223; lower: M = 6.44, SE = 0.223; *p* = 0.009), and right-side targets required fewer fixations than left-side targets (right: M = 5.85, SE = 0.223; left: M = 6.52, SE = 0.223; *p* < 0.001). These results indicate that the upper-right advantage observed in RT was also reflected in gaze allocation. The collapsed directional patterns are shown in Figure 9.

Overall, the eye-tracking results were consistent with the RT findings for the present VR-like menu stimuli. The gallery-based layout showed its most robust advantage in the upper-right quadrant, where it reduced both visit duration and fixation count relative to the grid-based layout. For TVD, an additional gallery-based advantage was observed in the lower-left quadrant.

### 4.4. Gaze-Distribution Analysis

**Figure 10:**
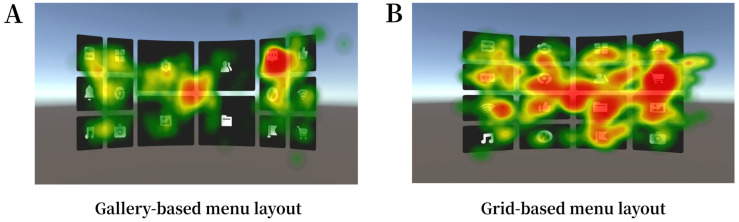
Gaze-distribution heatmaps over representative interface stimuli with the target located in the upper-right quadrant. (**A**) Gallery-based layout and (**B**) grid-based layout. Warmer colors indicate higher fixation density. The heatmaps provide qualitative visualizations of gaze allocation for the two layout structures under the same target-location condition.

Gaze-distribution heatmaps were generated as complementary visualizations of spatial attention allocation under the two menu layout structures. To provide a direct visual comparison between layouts, Figure 10 presents gaze-distribution heatmaps over a representative interface stimulus with the target located in the upper-right quadrant, where the most consistent layout-related advantage was observed in the RT, TVD, and TFC analyses. As shown in Figure 10, the grid-based layout exhibited relatively dispersed fixation density across multiple interface regions, indicating broader visual exploration during search. By contrast, the gallery-based layout showed more concentrated gaze clusters around a smaller number of visually salient regions. Under this simulated condition, this qualitative pattern was broadly consistent with the statistical results, in which the gallery-based layout produced shorter RTs, shorter visit durations, and fewer fixations than the grid-based layout in the upper-right quadrant.

**Figure 11:**
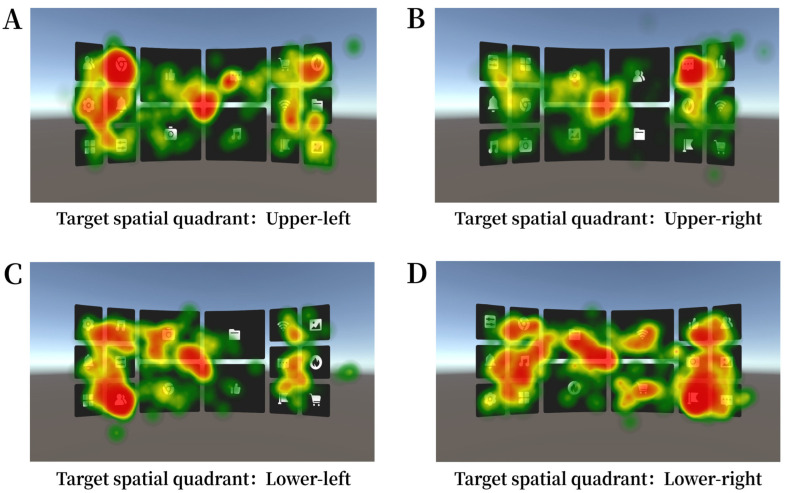
Gaze-distribution heatmaps over representative gallery-based menu interface stimuli with targets located in different spatial quadrants. (**A**) Upper-left; (**B**) upper-right; (**C**) lower-left; and (**D**) lower-right. Warmer colors indicate higher fixation density. The heatmaps are shown as qualitative examples to illustrate quadrant-related differences in gaze allocation within the gallery-based layout.

Figure 11 presents quadrant-level heatmaps using the gallery-based layout as an illustrative example. The heatmaps provide qualitative visual context for the spatial-location effects observed in the behavioral and eye-tracking analyses. Although fixation density was distributed across multiple interface regions in all conditions, the upper-right condition showed relatively compact high-density regions near the target-related area, whereas the upper-left and lower-left conditions showed broader gaze dispersion. These visual patterns should be interpreted qualitatively and were broadly consistent with the statistical results.

## 5. Discussion

### 5.1. Effects of Layout Structure on Visual Search Efficiency

This study examined whether card-based layout structure affects visual search efficiency in simulated VR menu interfaces. Overall, the gallery-based layout supported more efficient search than the grid-based layout, as reflected in shorter RTs, shorter overall TVD, and lower overall TFC. These results indicate that layout structure functions not only as a visual presentation format, but also as a perceptual factor that shapes attention allocation during menu search.

This advantage can be interpreted in terms of size-based visual hierarchy. In the gallery-based layout, larger cards may have served as perceptual anchors within the interface. Compared with the grid-based layout, where all cards were visually equivalent and arranged in a uniform matrix, the gallery-based layout provided differentiated visual regions that may have helped users organize the search space more efficiently. Such a pattern is consistent with classic and contemporary accounts of visual search, which suggest that attention can be guided by perceptual features, stimulus salience, and task goals [26,45,46]. In the present study, size variation may have increased the perceptual salience of certain regions, thereby reducing uniform scanning across all menu cards and supporting more efficient gaze allocation.

The gaze-based measures provide converging evidence for this explanation. TVD reflects the cumulative duration of visual attention allocated to the interface during search, whereas TFC reflects the frequency of visual sampling. The overall shorter TVD and lower TFC observed under the gallery-based layout indicate that participants required less sustained visual attention and fewer visual samples to complete the search task. This is consistent with eye-tracking research showing that fixation-based measures can provide useful indicators of attention allocation, search effort, and usability-relevant processing in human–computer interaction contexts [33,47,48,49,50].

This result complements prior VR menu research that has focused more on menu embodiment, interaction techniques, and selection methods. For example, Wang et al. [2] examined menu selection in immersive virtual environments by comparing fixed and handheld menus. Other recent VR studies have similarly emphasized gaze-, head-, and controller-based interaction techniques, selection efficiency, and user experience [10,11,18,19]. In contrast, the present study isolated card layout structure as a perceptual design factor in simulated VR menu interfaces. The findings suggest that even when the interaction mode is held constant, layout hierarchy itself can shape visual search efficiency and gaze allocation.

This finding should not be taken to imply that gallery-based layouts are universally superior in all VR menu contexts. The grid-based layout may still be appropriate when all menu items have equivalent priority or when systematic browsing is required. In contrast, gallery-based layouts may be more suitable for interfaces in which certain functions, categories, or content groups need to be visually prioritized. Thus, the main implication is not that one layout should always replace the other, but that size-based hierarchy can be used strategically to reduce visual search effort when rapid target localization is required.

### 5.2. Spatial Asymmetries in VR Menu Search

The results also revealed systematic spatial asymmetries in visual search performance. Target spatial quadrant significantly influenced RT, TVD, and TFC, indicating that search efficiency was not spatially uniform across the menu interface. In particular, targets located in the upper-right quadrant showed the fastest responses, and the eye-tracking results showed a broadly consistent reduction in gaze-based search effort, especially under the gallery-based layout. Directional follow-up analyses further showed that upper targets were associated with faster responses than lower targets, and right-side targets showed the same advantage over left-side targets. Similar patterns were observed in the eye-tracking measures, with upper and right-side targets associated with shorter visit durations and fewer fixations.

These findings suggest that the upper-right advantage was not an isolated quadrant-level effect but rather reflected the combined contribution of upper-region and right-side facilitation. This pattern is compatible with the broader literature on spatial heterogeneity in visual attention and visual-field performance. Carrasco [27] reviewed extensive evidence showing that spatial attention plays a central role in early visual processing and selective information extraction. Earlier work on visual-field specialization suggested that visual performance varies across upper and lower visual fields [28,34]. Recent studies further indicate that visual performance fields are spatially heterogeneous and can be modulated by polar angle, task demands, and measurement context [51,52].

At the same time, the upper-right advantage observed in the present study should not be interpreted as a direct replication of low-level visual-field asymmetries. In many basic perceptual tasks, performance differences across the visual field can depend on meridian, eccentricity, stimulus type, and task demands [12,29,52,53]. The present task involved structured menu search rather than isolated low-level detection, and therefore likely involved both bottom-up and top-down factors. In interface environments, spatial asymmetry may be shaped by visual-field constraints, reading habits, learned scanning routines, interface conventions, and task goals [31,32].

This interpretation also helps distinguish the present icon-search task from text-heavy web browsing contexts, where usability studies have described F-shaped scanning patterns as a typical pattern of page reading or content browsing [54,55]. Because participants in the present study localized target icons within a spatially distributed VR-like card menu rather than reading sequential text, the upper-right advantage should be interpreted as task- and layout-dependent rather than as a contradiction of left-biased scanning in conventional web interfaces.

In this context, the right-side advantage observed here is also consistent with recent evidence that right-visual-field advantages can emerge in controlled perceptual tasks and may be modulated by attentional balance or task context [13,30]. In VR and 3D interaction contexts, prior work has also shown that interaction efficiency can differ across visual areas [35], suggesting that spatial placement may remain relevant even when the interface departs from conventional 2D page layouts. Therefore, the present findings suggest that target location is not a neutral design factor in VR-like menu search. Spatial placement can influence both behavioral efficiency and gaze allocation, especially when rapid target localization is required.

### 5.3. Joint Effects of Layout Structure and Target Spatial Quadrant

A key finding was the significant interaction between menu layout structure and target spatial quadrant, indicating that the layout effect varied across spatial locations. The advantage of the gallery-based layout was therefore spatially dependent rather than uniform across the interface.

The most robust layout-related advantage emerged in the upper-right quadrant. In this quadrant, the gallery-based layout produced significantly shorter RTs, shorter TVD, and lower TFC than the grid-based layout. This convergence across behavioral and eye-tracking measures suggests that the upper-right region provided a condition in which layout hierarchy and spatial facilitation were mutually reinforced. In other words, the size-based visual hierarchy of the gallery-based layout may have guided attention more efficiently when the target was located in a spatial region that was already favorable for rapid search.

The TVD results also showed an additional gallery-based advantage in the lower-left quadrant, although this effect was not consistently reflected in RT and TFC. This pattern suggests that the gallery-based layout may reduce sustained visual processing in some relatively demanding spatial regions, even when this reduction does not fully translate into faster responses or fewer fixations. This partial divergence may reflect the different aspects of visual search captured by the three measures. RT reflects the final behavioral response and includes not only visual search, but also target confirmation, left/right decision-making, and motor response. TFC reflects the number of visual sampling steps, whereas TVD captures the cumulative duration of gaze allocation and may be more sensitive to sustained attentional processing. Thus, in the lower-left quadrant, the gallery-based layout may have shortened the duration of attentional processing without clear evidence of reducing the number of fixations or the overall response time. Therefore, the lower-left result should be interpreted as a metric-specific effect rather than as a comprehensive gallery-layout advantage.

These findings highlight the importance of considering layout structure and spatial location together. Interface layout optimization cannot be reduced to a global preference for one visual arrangement over another. A layout that improves search efficiency in one region may have weaker or different effects in another. This pattern is consistent with salience-based and guided-search accounts, which suggest that visual search is shaped by stimulus-driven salience and task goals [26,46]. From an applied attention perspective, the effectiveness of a visual layout may also depend on task context and information placement [56]. For VR menu design, this means that layout hierarchy and target placement should be jointly considered, especially when arranging high-priority or frequently accessed functions.

The interaction result also extends previous interface search studies in which layout, visual grouping, icon properties, or information organization were shown to affect search efficiency and gaze behavior [6,7,8,9,24]. The present study adds to this evidence by showing that, in simulated VR menu interfaces, layout hierarchy and target spatial quadrant can jointly shape visual search performance. This suggests that VR interface layout should be evaluated not only at the global layout level, but also in relation to where task-relevant information is placed.

### 5.4. Design Implications and Eye-Tracking-Based Evaluation Value

The present findings offer practical implications for early-stage VR menu interface design and simulated interface evaluation. From a design perspective, size-based hierarchy can be used to create perceptual anchors when rapid visual search is required. In card-based VR menus, visually prominent cards may help structure the search space and guide attention toward task-relevant regions. This strategy may be particularly useful when menus contain functions or content categories with different levels of priority.

The results also suggest that spatial placement should be considered together with visual hierarchy. The upper-right advantage observed in this study indicates that, in similar menu-search contexts, high-priority or frequently accessed functions may benefit from being placed in upper or right-side regions, particularly when combined with a layout structure that provides clear visual organization.

Beyond design guidance, the present findings also demonstrate the value of screen-based eye tracking for early-stage evaluation of simulated VR menu layouts. RT provided a behavioral index of whether one layout supported faster target localization, whereas TVD and TFC helped reveal how layout structure influenced gaze allocation and search effort. In this study, the gallery-based layout not only shortened RTs but also reduced overall visit duration and fixation count, suggesting lower gaze-based search effort. The heatmap analysis further provided qualitative visual context: in the representative upper-right stimulus, gaze allocation appeared more concentrated in the gallery-based layout, whereas the grid-based layout showed broader visual exploration. Taken together, these results suggest that screen-based eye tracking can provide a controlled and reproducible way to compare layout alternatives before more complex VR implementation. This early-stage approach can be further complemented by later HMD-based studies incorporating head-motion sensing, stereoscopic depth, and more naturalistic interaction techniques.

### 5.5. Limitations and Future Work

Several limitations should be acknowledged when interpreting the present findings. The main limitation concerns the use of a desktop-based VR simulation rather than a fully immersive HMD-based VR environment. Although the stimuli preserved the spatial organization and shallow curved presentation of VR menu panels, the desktop-based setting did not capture all perceptual and interaction characteristics of immersive VR, such as stereoscopic depth perception, head movement, head–eye coordination, gaze-based selection, controller-based pointing or selection, and embodied interaction. In particular, the present setup did not fully account for head-orientation adjustments that users may naturally make to bring targets closer to the center of view in immersive VR, which may influence visual search strategies. Therefore, the findings should be interpreted as early-stage perceptual evidence rather than direct evidence for fully immersive VR interaction performance. Because immersion level can influence user experience and interaction behavior in virtual environments [57,58], future studies should validate these findings in HMD-based VR systems with integrated eye tracking and head-motion sensing.

The experimental task also involved static rendered screenshots and a simplified visual search response. Although this design was suitable for isolating layout-driven perceptual effects, real VR menu interaction often includes dynamic feedback, hand or controller input, sequential navigation, and multimodal cues. Future work should therefore examine whether the observed layout and spatial-location effects persist under more interactive conditions, such as gaze-based selection, controller pointing, or hand-tracking input [59].

Another limitation relates to the range of layout structures examined. The study compared two representative card-based layouts: a uniform grid-based layout and one representative gallery-based layout with size-based hierarchy. The gallery-based layout tested here was a specific configuration consisting of four large cards and twelve small cards with fixed large-card positions. Therefore, the present findings should be understood as evidence from one representative size-hierarchical gallery-style configuration. They may offer useful design reference for similar card-based VR menu layouts, while future parametric studies are needed to examine how well the effects extend to other gallery variants or VR menu configurations. In addition, the broader literature on 3D and VR menu techniques has identified a wide range of menu forms and interaction techniques, such as radial menus, depth-layered menus, and other spatial menu structures [60]. These broader menu forms were beyond the scope of the present study. Future research should extend the comparison to a wider range of VR menu structures and examine how layout geometry, depth arrangement, information density, card-size ratio, number of emphasized cards, card spacing, large-card placement, and visual salience jointly affect search efficiency.

The participant sample and gaze metrics should also be considered. Participants were mainly university students, and users with different VR experience, age, visual ability, or professional backgrounds may adopt different search strategies. In addition, the present study focused on TVD, TFC, and heatmaps as primary gaze-based indicators. Future studies could include more diverse user groups and combine these measures with scan-path metrics, such as transition probability, scan-path entropy, or gaze-sequence similarity, to provide a more detailed account of visual search behavior.

Taken together, these limitations indicate that the present findings should be further validated in more immersive VR settings, with broader menu structures and more naturalistic interaction techniques. Within this scope, the study provides early-stage perceptual evidence that layout structure and target spatial quadrant may jointly influence visual search efficiency in simulated VR menu interfaces, and that eye-tracking metrics can support early evaluation of attention allocation during interface optimization.

## 6. Conclusions

This study used a desktop-based VR simulation combined with screen-based eye tracking to examine how menu layout structure and target spatial quadrant influence visual search efficiency in VR-like menu interfaces. By comparing a uniform grid-based card layout with a gallery-based card layout containing size-based visual hierarchy, the study evaluated behavioral performance, indexed by reaction time (RT), together with sensor-derived gaze measures, including total visit duration (TVD), total fixation count (TFC), and gaze-distribution heatmaps. The results showed that the gallery-based layout supported more efficient visual search than the grid-based layout, as reflected in shorter RTs, reduced overall TVD, and lower overall TFC. Target spatial quadrant also influenced search performance, with the upper-right quadrant showing the fastest responses; further analyses indicated that upper and right-side targets were associated with faster responses and lower gaze-based search effort. Importantly, the gallery-based layout showed its most consistent advantage in the upper-right quadrant, indicating that layout hierarchy and target placement jointly shaped visual search efficiency. These findings provide early-stage empirical evidence for evaluating layout alternatives in simulated VR-like menu interfaces and demonstrate that eye-tracking sensors can provide objective, interpretable, and fine-grained measurements of attention allocation during early-stage VR interface evaluation. Future studies should validate these results in fully immersive VR environments with integrated HMD-based eye tracking, head-motion sensing, more naturalistic interaction tasks, and a wider range of menu structures.

## Figures and Tables

**Table 1:** Means and standard deviations of accuracy and reaction time (RT, s) across experimental conditions.

Menu Layout Structure	Target Spatial Quadrant	ACC		RT	
		Mean	SD	Mean (s)	SD
Gallery-based	LL	0.991	0.092	1.498	0.615
	LR	0.974	0.159	1.618	0.759
	UL	0.974	0.159	1.571	0.969
	UR	1.000	0.000	1.178	0.449
Grid-based	LL	1.000	0.000	1.710	0.666
	LR	0.983	0.130	1.517	0.621
	UL	0.974	0.159	1.697	0.840
	UR	1.000	0.000	1.481	0.573

Note. LL = lower-left; LR = lower-right; UL = upper-left; UR = upper-right.

**Table 2:** Bonferroni-corrected simple-effect comparisons of RT between layout structures within each target spatial quadrant.

Target Spatial Quadrant	Contrast	Estimate (s)	SE	t	*p*_adj
LL	Gallery-based − Grid-based	−0.203	0.092	−2.209	0.110
LR	Gallery-based − Grid-based	0.112	0.094	1.185	0.946
UL	Gallery-based − Grid-based	−0.110	0.094	−1.166	0.976
UR	Gallery-based − Grid-based	−0.313	0.092	−3.391	0.003

Note. Negative estimates indicate shorter RTs in the gallery-based layout than in the grid-based layout. *p*_adj values were Bonferroni-corrected across the four quadrant-specific layout comparisons.

**Table 3:** Eye-tracking descriptive statistics across menu layout structures and target spatial quadrants.

Menu Layout Structure	Target Spatial Quadrant	TVD		TFC	
		Mean (s)	SD	Mean	SD
Gallery-based	LL	1.245	0.534	6.200	2.904
	LR	1.334	0.706	6.520	2.997
	UL	1.298	0.857	6.194	3.994
	UR	0.987	0.437	4.840	2.078
Grid-based	LL	1.456	0.603	6.962	2.923
	LR	1.265	0.535	6.172	2.778
	UL	1.447	0.716	6.842	3.313
	UR	1.238	0.522	6.093	2.504

**Table 4:** Bonferroni-corrected simple-effect comparisons of eye-tracking measures between layout structures within each target spatial quadrant.

Measure	Target Spatial Quadrant	Contrast	Estimate	SE	t	*p*_adj
TVD	LL	Gallery − Grid	−0.200	0.078	−2.565	0.042
	LR	Gallery − Grid	0.079	0.080	0.987	1.000
	UL	Gallery − Grid	−0.115	0.080	−1.439	0.602
	UR	Gallery − Grid	−0.277	0.078	−3.537	0.002
TFC	LL	Gallery − Grid	−0.736	0.385	−1.913	0.224
	LR	Gallery − Grid	0.359	0.395	0.910	1.000
	UL	Gallery − Grid	−0.531	0.394	−1.348	0.712
	UR	Gallery − Grid	−1.339	0.386	−3.469	0.002

Note. Negative estimates indicate lower values in the gallery-based layout than in the grid-based layout. *p*_adj values were Bonferroni-corrected across the four quadrant-specific layout comparisons for each measure.

## Data Availability

The original contributions presented in this study are included in the article. Further inquiries can be directed to the corresponding author.
